# IRF-1 regulates alternative mRNA splicing of carcinoembryonic antigen-related cell adhesion molecule 1 (CEACAM1) in breast epithelial cells generating an immunoreceptor tyrosine-based inhibition motif (ITIM) containing isoform

**DOI:** 10.1186/1476-4598-13-64

**Published:** 2014-03-21

**Authors:** Kenneth J Dery, Maciej Kujawski, David Grunert, Xiwei Wu, Tung Ngyuen, Celeste Cheung, John H Yim, John E Shively

**Affiliations:** 1 Departments of Immunology, Beckman Research Institute, City of Hope, Duarte, California, USA; 2 Molecular Medicine, Beckman Research Institute, City of Hope, Duarte, CA, USA; 3 Surgery, Beckman Research Institute, City of Hope, Duarte, CA, USA

**Keywords:** CEACAM1, CEACAM1-L, IRF-1, IFN-γ, ISRE, Alternative splicing, Inflammation

## Abstract

**Background:**

Interferon regulatory factor-1 (IRF-1) is a master regulator of IFN-γ induced gene transcription. Previously we have shown that IRF-1 transcriptionally induces CEACAM1 via an ISRE (Interferon-Stimulated Response Element) in its promoter. CEACAM1 pre-mRNA undergoes extensive alternative splicing (AS) generating isoforms to produce either a short (S) cytoplasmic domain expressed primarily in epithelial cells or as an ITIM-containing long (L) isoform in immune cells.

**Methods:**

The transcriptional and molecular mechanism of CEACAM1 minigenes AS containing promoter ISREs mutations in the breast epithelial, MDA-MB-468, cell line was detected using flow cytometry. In addition, transcriptome sequencing was utilized to determine whether IRF-1 could direct the AS of other genes as well. Tumor xenografts were used to evaluate CEACAM1 isoform expression on the leading edge of breast tumor cells.

**Results:**

In the present study, we provide evidence that CEACAM1’s promoter and variable exon 7 cross-talk allowing IRF-1 to direct AS events. Transcriptome sequencing shows that IRF-1 can also induce the global AS of genes involved in regulation of growth and differentiation as well as genes of the cytokine family. Furthermore, MDA-MB-468 cells grown as tumor xenografts exhibit an AS switch to the L-isoform of CEACAM1, demonstrating that an *in vivo* inflammatory milieu is also capable of generating the AS switch, similar to that found in human breast cancers Mol Cancer 7:46, 2008.

**Conclusions:**

The novel AS regulatory activities attributed to IRF-1 indicate that the IFN-γ response involves a global change in both gene transcription and AS in breast epithelial cells.

## Background

Interferons (IFNs) are a group of immunomodulatory proteins made and released by host cells in response to the presence of pathogens such as viruses, bacteria, parasites or tumor cells. IFNs are primarily known for their role in inhibiting viral infections and in stimulating the immune system [1]. IFN-γ, a type II interferon, is a pleiotropic cytokine involved in the regulation of immune and inflammatory responses, including the activation, growth and differentiation of B-cells, T-cells, natural killer (NK) cells and macrophages. Aberrant IFN-γ expression, associated with autoinflammatory and autoimmune diseases, acts as a pro-inflammatory cytokine [2]. IFN-γ is produced predominantly by natural killer and natural killer T cells as part of the innate immune response, and by CD4 Th1 and CD8 cytotoxic T lymphocyte effector T cells once antigen-specific immunity develops [3].

IFN-γ induces the JAK/STAT pathway leading to the activation and binding of transcriptional activators that induce expression of IFN-stimulated genes, notably IRF-1. IRF-1 is expressed at low levels in unstimulated cells and recognizes the ISRE found in most IFN-inducible gene promoters containing repeating short GAAA core sequences [4,5]. We have previously shown that CEACAM1 transcription can be induced by IFN-γ via an ISRE in the CEACAM1 promoter [6-8]. CEACAM1 is a member of the immunoglobulin super family of glycoproteins [9]. It is expressed on the surface of epithelial and activated endothelial cells, as well as on cells from the immune system and plays a role in a variety of cellular processes including cell-cell adhesion, differentiation, apoptosis and immune response [10].

In humans and rat, CEACAM1 pre-mRNA undergoes extensive AS generating isoforms consisting of an N-terminal and a variable number of multiple extracellular Ig-like domains, a transmembrane domain, and either short (CEACAM1-S) or long (CEACAM1-L) cytoplasmic domains [11-13]. The short cytoplasmic domain isoform, the predominant isoform in epithelial cells, has been shown to bind actin, tropomyosin, calmodulin, and annexin II and is involved in lumen formation [14-16]. In normal breast, it is the predominant isoform, while in breast cancer the S/L ratio is greatly reduced [17]. The long cytoplasmic domain isoform with its two cytosolic phosphotyrosine residues and immunoreceptor tyrosine-based inhibitory motifs (ITIM), predominant in immune cells, bind SHP-1 when phosphorylated and convey inhibitory activities to CEACAM1-L [18]. Furthermore, in T lymphocytes CEACAM1-L isoforms are significantly upregulated at the cell surface in response to IL-2 treatment, mediating their cell adhesion to other lymphocytes or tumor cells [19,20].

The observation that CEACAM1 functions in recognition and binding between cells with adhesion properties is integral to its signal transduction leading to growth suppressive activities [21]. Indeed, CEACAM1 is a tumor suppressor gene that is down-regulated in most tumor cells such as colon, breast and prostrate cancer [21-23]. Introduction of CEACAM1-L but not CEACAM1-S cDNA by adenovirus as a gene delivery vehicle reduces tumorigenicity of breast cancer cells [21]. These findings are consistent with the idea that the expression of CEACAM1 plays a role in keeping cells in a differentiated state, while its down-regulation may be an essential event in the development of epithelial malignancy [24]. However, the implication of the regulation of its AS generated isoforms is less well understood.

AS is a genomic evolutionary strategy that generates proteome expansion in metazoans [25,26]. *cis-*Regulatory elements known as exonic splicing enhancers and exonic splicing silencers (ESS ) provide the binding platform for splicing regulators to promote or inhibit the use of nearby splice-sites (ss). AS effectors include members of the heterogeneous nuclear ribonucleoprotein (hnRNP) family and serine/arginine-rich (SR) protein families are among the most common splicing regulators [27,28]. hnRNPs rapidly associate with nascent transcripts and repress certain AS events leading to exon skipping events [25]. Three abundant hnRNPs (hnRNP L, hnRNP A1, and hnRNP M) are implicated in many AS events in human and several other eukaryotes and play a role in regulating splicing events by inhibiting the use of splice-sites from the spliceosome [11]. Previously we showed that hnRNP L and hnRNP A1 interact specifically with exon 7 to control splice-site recognition in the formation of CEACAM1-S mRNA. hnRNP M is important for the AS of CEACAM1-L mRNA [11].

We recently showed that IFN-γ altered the ratio of splice isoforms of CEACAM1 through the presumable induction of its down-stream effector IRF-1 [8]. The goal of the present study was to determine whether forced expression of IRF-1 directly plays a role in regulating CEACAM1 AS. Using GFP-driven reporter constructs containing the CEACAM1 genomic promoter and its exons 6-7-8 (heretofore called CAM1 6-7-8) cassettes, we show that AS depends on IRF-1 to couple transcriptional complex communication with the splicing machinery. Transcriptome sequencing analysis shows that IRF-1 can induce the AS of other genes as well, including those involved in membrane regeneration and repair, microtubule assembly, and tumor suppressor functions. We also report extensive changes in transcriptional and AS regulation of cytokines in breast cancer epithelial cells in response to IRF-1 treatment. The AS regulatory activities of IRF-1 demonstrate that the mechanism of the interferon response includes a global change in both gene transcription and AS in breast epithelial cells.

## Results

### Ad-IRF-1 treatment induces the CEACAM1-L isoform in a breast cancer line

Although normal breast epithelial cells predominantly express the CEACAM1-S isoform, we have observed a switch of the ratio of S/L isoforms in favor of the L isoform in breast cancer by an unknown mechanism [17]. More recently, while investigating changes in transcription factor binding to the CEACAM1 promoter in MDA-MB-468 breast cells after treatment with IFN-γ, we observed the induction of CEACAM1-L isoform suggesting that the AS mechanism involves the ISRE of CEACAM1’s promoter [8]. Since the ISRE involves binding of IRF-1, we investigated if forced expression of IRF-1 could directly generate the L isoform.

**Figure 1:**
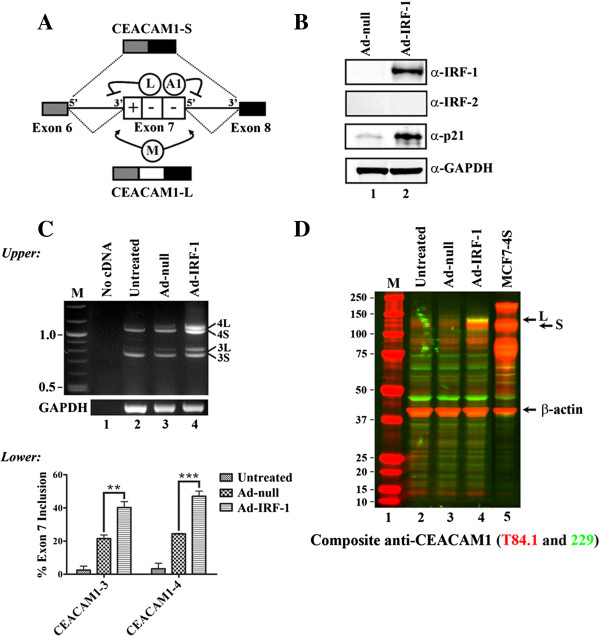
**Forced expression by Ad-IRF-1 generates CEACAM1-L isoform in MDA-MB-468 breast carcinoma cells. (A)** Model of *cis* and *trans*-acting elements involved in CEACAM1 AS. A general splicing mechanism involves physical interaction of hnRNP L and hnRNP A1 (enclosed in circles) with exon 7 (indicated by white box) at ESSs (indicated by – sign) to generate CEACAM1-S through repression of 3’ and 5’ ss (inhibitory arrows). hnRNP M promotes formation of CEACAM1-L through exon definition (arrows) as part of the regulatory splicing complex that participates with the Exon Splicing Enhancer (ESE; + sign). **(B)** Total cellular protein lysates from viral vector treated (Ad-null) or Ad-IRF-1 treated cells were subjected to Western blot and probed with antibodies to IRF-1, IRF-2, and p21. GAPDH was used as a loading control. **(C)** Induction of isoform CEACAM1-3L and CEACAM1-4L mRNA by Ad-IRF-1 after 24 h. (*Upper*) Total RNAs were isolated, subjected to RT-PCR and separated by electrophoresis on a 2.25% agarose gel. The position of CEACAM1-3 and CEACAM1-4 splice variants are indicated on right. M refers to a 100-bp DNA ladder (New England Biolabs). (*Lower)* Histogram quantification of data is n =3 independent experiments. The mean ± S.E. shown for percent exon 7 inclusion was calculated as the % [CEACAM1-L/(CEACAM1-L + CEACAM1-S)] mRNA. **, p < 0.01; ***, p < 0.001 versus Ad-null control. **(D)** Western blotting of cell lysates after induction of Ad-IRF-1 after 24 h with antibody T84.1 (red channel) which recognizes both isoforms of CEACAM1 (-S and –L) and antibody 229 (green channel) which recognizes CEACAM1-L isoform exclusively (each individually found in Additional file 3: Figure S3A-B). Shown is a composite of superimposed antibodies to highlight production of CEACAM1-L (yellow combined channels) after Ad-IRF-1 treatment.

The AS of CEACAM1 is dependent on splicing regulatory domains in exon 7 [11]. These *cis*-elements are capable of recruiting various trans-regulatory proteins to direct splice-site recognition involved in exon 7 definition. Specifically, exon 7 contains two distinct ESSs that recruit hnRNP L and hnRNP A1 to direct exon-skipping by the spliceosome whereas hnRNP M leads to exon-inclusion, in the production of the L-isoform (Figure 1A).

Ectopically expressed IRF-1 in the breast carcinoma cell line MDA-MB-468 has been previously described using a recombinant adenovirus (Ad-IRF-1) as a gene transfer vehicle containing the mouse IRF-1 gene under a constitutive, cytomegalovirus promoter [29,30]. Using this system, we observed the expression of IRF-1 but not IRF-2, a repressor of CEACAM1 transcription and other target genes [31], 12 h after infection with an apparent peak at 24 h (Figure 1B and Additional file 1: Figure S1A). IRF-1 expression was absent in the Ad-null control-infected cells. The induction of the cyclin-dependent kinase inhibitor p21 (WAF1/CIP1) was included to demonstrate that effector IRF-1 was functionally competent [32].

We next addressed whether Ad-IRF-1 in MDA-MB-468 cells could replicate IFN-γ-directed AS of CEACAM1. CEACAM1-4L and –S mRNA isoforms, but not CEACAM1-3L and -3S isoforms, are strongly up-regulated by Ad-IRF-1 as compared to virus vector control (Figure 1C, lane 4 versus lane 3). CEACAM1-4L and CEACAM1-4S contain four extracellular immunoglobulin-like domains while CEACAM1-3L and CEACAM1-3S only three. Notably, in both isoforms we detected strong activation of spliceosome processing (more than 2-fold) of exon 7 containing mRNA transcripts between 12-24 h post infection (CEACAM1-4L, p < 0.001 and CEACAM1-3L, p < 0.01; Figure 1C). We next tested MCF7 cells, derived from breast adenocarcinoma, with Ad-IRF-1 treatment to see if we could detect a similar AS phenotype (Additional file 2: Figure S2A-B). We did not detect evidence of CEACAM1 mRNA using quantitative real-time PCR analyses in untreated cells consistent with previous observations [14]. By contrast, stimulation of cells by Ad-null treatment caused a significant and strong up-regulation of CEACAM1-S but not CEACAM1-L mRNA as early as 6 h post-treatment. We also observed that IRF-1 transcriptionally induces more CEACAM1-S but not CEACAM1-L mRNA at all time points tested. Only at 24 h we observed the emergence of CEACAM1-L mRNA, albeit approximately 1000-fold less. This is in contrast to our observations in MDA-MB-468 cells that production of the L-isoform starts as early as 6 hrs after Ad-IRF-1 treamment (Additional file 1: Figure S1B). In the case of the MCF7 cell line response to Ad-null viral infection, it is likely that an additional immune response is generated unrelated to splicing; e.g., a TLR9 response could account for this.

Total lysates from treated and untreated Ad-IRF-1 cells were next subjected to Western blots to establish correlative protein to RNA expression levels (Figure 1D and Additional file 3: Figure S3A-B). Using antibody 229 (which detects CEACAM1-L; green channel) and T84.1 (which detects both CEACAM1 isoforms, red channel) and fluorescent secondary antibodies we were able to distinguish between low levels of CEACAM1-S in untreated and viral control cells and stimulated production of CEACAM1-L after Ad-IRF-1 treatment (yellow band, Figure 1D, lane 4). After Ad-IRF-1 treatment, protein levels of CEACAM1-S increased over basal levels by Western blot and FACS analysis (Figure 1D, lane 4 and Additional file 4: Figure S4). We conclude that a novel mechanism for IRF-1 function has been uncovered and it involves post-transcriptional AS.

### An ISRE with a GG->CC substitution disrupts IRF-1 binding

**Figure 2:**
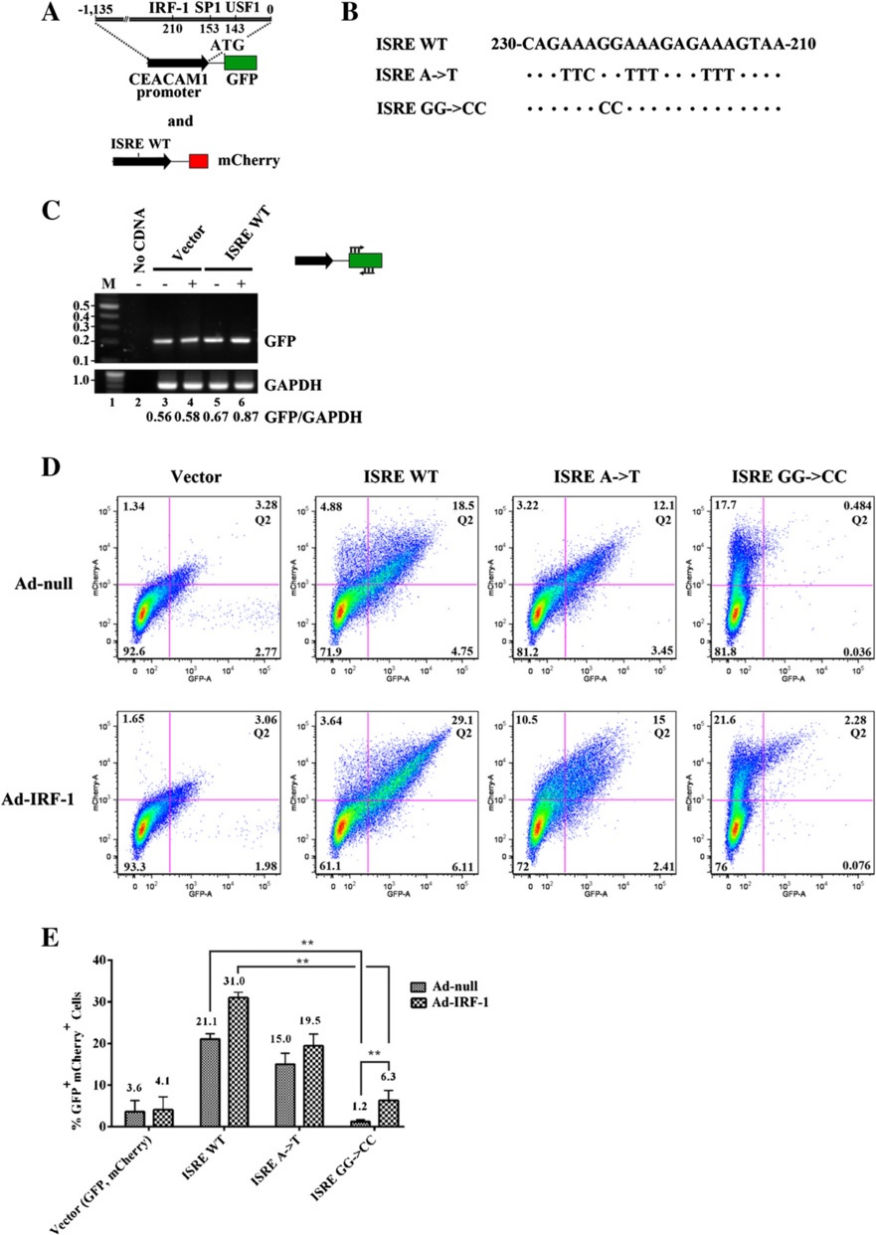
**ISRE GG->CC mutant impairs GFP expression in CEACAM1 promoter-driven constructs. (A)** Schematic diagram of the -1,135-kb fragment, in relation to the ATG start codon, of the CEACAM1 promoter (upper black arrow) fused in translational reading frame to GFP (green box). Known transcription factor binding sites to IRF-1, SP1 and USF-1 are indicated. A mCherry reporter that was co-transfected and used as a quantitation control was fused to CEACAM1 WT ISRE (lower black arrow). **(B)** Nucleotide comparisons of the 21-bp ISRE with mutant alleles (A->T and GG->CC). **(C)** RT-PCR of GFP gene expression. Total RNAs were isolated, subjected to semi-quantitative PCR and separated by electrophoresis on a 1% agarose gel. GFP and GAPDH are indicated to the right and primer positions for ISRE promoter-driven GFP are indicated in the schematic. **(D)** Flow cytometric analyses of CEACAM1 ISRE WT and mutant promoter alleles GFP reporter constructs after Ad-null or Ad-IRF-1 treatment showing mCherry (Y-axis) and GFP (X-axis). Results are from one of three experiments with similar results. **(E)** Histogram of Quadrant 2 (Q2) in Figure 2D shows % cells expressing vector, ISRE WT, or mutant ISREs in GFP and ISRE WT mCherry reporter activity (% GFP^+^ mCherry^+^ cells). Results are from n = 3 independent experiments and **, p < 0.01 versus ISRE WT controls are indicated.

To elucidate the molecular mechanism of IRF-1 dependent AS, we created ISRE mutants that contained purine to pyrimidine substitutions. Previously, it was shown that the major groove of IRF-1 contacts bases that localize to a well-established consensus GAAA sequence within the 13-bp PRD I element [5]. Examination of CEACAM1’s core ISRE shows three such motifs flanking a central 223-GG-224 dinucleotide (Figure 2B). We created mutant ISRE A->T by introducing AAA->TTT or TTC substitutions along the ISRE. Additionally, we previously reported an *in vivo* footprint in the ISRE (-223/4 nt relative to the TSS) that indicated 223-GG-224 bases interact directly with IRF-1 on the sense but not antisense strand [8]. Therefore, mutant ISRE GG->CC was created to test this target sequence.

We hypothesized that weakening IRF-1’s association with CEACAM1’s ISRE would inhibit AS events. To test this hypothesis, we first cloned WT and mutant ISREs (from a 1.135-kb promoter CEACAM1 DNA fragment relative to the TSS) in translational fusion to a GFP reporter (Figure 2A). We first tested whether our system was responsive to infection with Ad-IRF-1 using the ISRE WT construct and performed RT-PCR to determine whether we could detect a stable GFP-containing message (Figure 2C). Our data of IRF-1 treatment in ISRE WT compared to Vector alone demonstrates responsiveness to Ad-IRF-1 delivery [lane 6 (0.87) compared to lane 4 (0.58) GFP:GAPDH ratio]. These ISRE promoter-GFP constructs were next transiently transfected into MDA-MB-468 cells. To avoid misinterpretation of weak reporter activity resulting from potential loss-of-function phenotypes, we also co-transfected WT CEACAM1 ISRE fused to mCherry, as a transfection control (Figure 2A). Our dual reporter system provides in effect an *in vivo* flow-cytometry read-out of the efficacy of promoter mutations as a function of transfection efficiency, as previously shown [33]. As shown in Figure 2D, one of three independent experiments, and summarized in Figure 2E, mock-treated ISRE WT samples but not vector controls produced double positive GFP/mCherry cells (21.1% compared to 3.6%). Interestingly, we note that the percent double positive cells observed for the ISRE A->T mutant decreased by only 28.9% (15.0/21.1), whereas by comparison, the mutant ISRE GG->CC decreased by a potent 94.3% (1.2/21.1; p < 0.01). We found that the addition of exogenous IRF-1 increased reporter activity by almost 1.5-fold in the ISRE WT reporter as compared to the vector control which showed no significant difference. Notably, the target mutant ISRE GG->CC predicted by footprinting showed more than a 5-fold increase in GFP expression [6.3/1.2; p < 0.01] when presented with Ad-IRF-1 compared to the weaker result by the consensus mutant ISRE A->T. We next harvested and collected RNA from these cells and tested by RT-PCR whether we could detect changes in GFP expression at the level of RNA (data not shown). Consistent with our flow cytometry observations, we conclude that transcriptional activation of GFP by ISRE WT and ISRE GG->CC promoter is enhanced by the addition of Ad-IRF-1 (Figure 2E). We also conclude that no other transcriptional factors (SP1, USF1) substitute for IRF-1 when ISRE promoter association has been dysregulated (Figure 2A; see genetic organization of CEACAM1 promoter).

**Figure 3:**
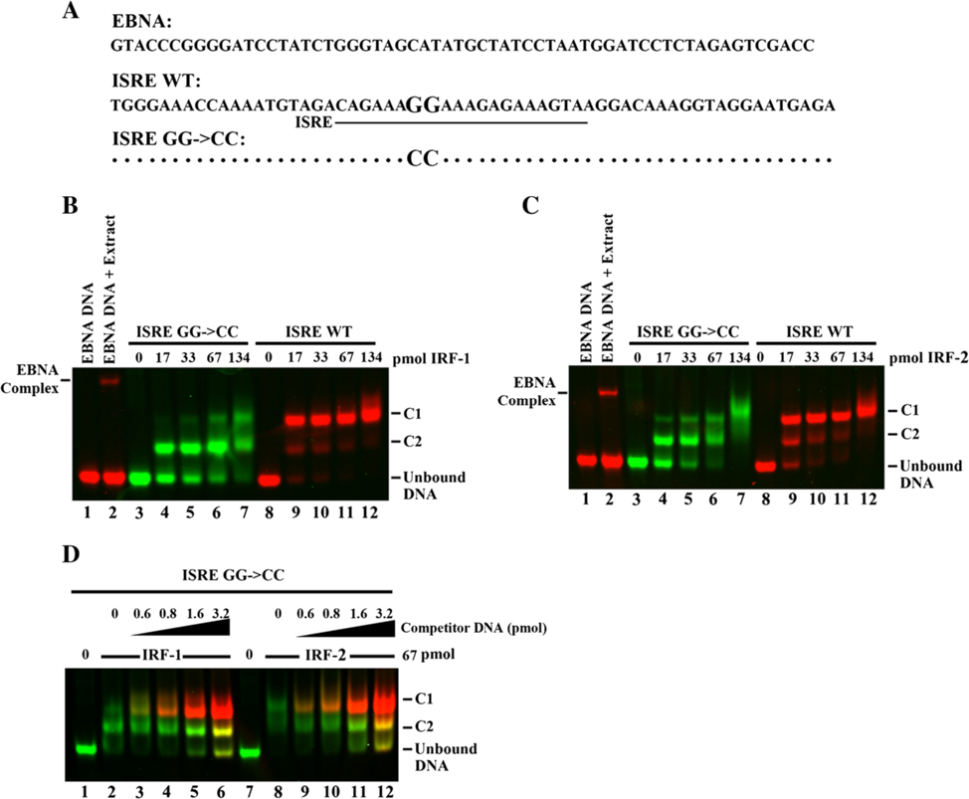
**GG->CC mutation in the CEACAM1 ISRE impairs IRF-1 and IRF-2 Binding. (A)** CEACAM1 ISRE (underline) containing 60-mer oligonucleotides are shown with enlarged nt (GG) in the WT or (CC) in the mutant. EBNA DNA served as the positive control for binding. **(B-D)** Fluorescent electrophoretic mobility shifts (fEMSA) were performed with 0.3 pmol IRDye-labeled oligonucleotides (EBNA-700 nm, ISRE GG->CC-800 nm and ISRE WT-700 nm) and increasing moles of IRF-1 **(B)** or IRF-2 **(C)** as shown. Epstein-Barr Nuclear Antigen extract is used to form a positive control complex denoted by EBNA complex (left). DNA-protein complexes produced by incubation with specific IRF proteins produce either a slow-migrating (C1) or faster-migrating complex (C2). **(D)** Competition assays were performed by adding increasing amounts of ISRE-WT DNA (red channel) in the presence of 0.3 pmol ISRE GG->CC DNA (green channel) and either 67 pmol IRF-1 or IRF-2 protein. Addition of competitor DNA to produce all three binding states (unbound, yellow C2 and C1 complexes) indicates complete saturation of exogenous IRF protein. Results are from one of three experiments with similar results.

To characterize the high inhibition observed for the ISRE GG->CC promoter mutant, we next performed a fluorescent Electromobility Shift Assay (fEMSA) using IRDye (infrared dye)-labeled 60-mer oligonucleotides (Figure 3A). We chose the Epstein-Barr virus internal ribosome entry site (EBNA) sequence to serve as our positive control for EMSA binding. From the data presented we show that the binding affinity for IRF-1 by the ISRE GG->CC mutant (green channel) is significantly impaired when compared to the ISRE WT sequence (Figure 3B, lanes 4-7 versus lanes 9-12; red channel). At the lowest ratio of IRF-1 to ISRE DNA (17:0.3), we observed the majority of ISRE WT DNA formed a slow migrating IRF-1 containing complex (denoted C1 in the figure) and a faster migrating complex (denoted C2; lane 9). By contrast, the ISRE GG->CC mutant made no C1 complex, while 50% of the bound DNA was in the C2 complex and another 50% remained in an unbound state, suggesting that the dinucleotide mutation caused a weakening of IRF-1 binding (lane 4). Since complex formation still occurs in the mutant, it suggests that CEACAM1’s ISRE containing repeat-short GAAA sequences still play an important role in binding [5].

We also tested whether IRF-2 binding is affected by the target mutant ISRE GG->CC. Previous studies have shown that IRF-2 competitively inhibits IRF-1 by binding to the same regulatory elements causing inhibition of IRF-1-mediated transcriptional activation of IFN-inducible genes [34]. As shown in Figure 3C, fEMSAs performed with IRF-2 and the ISRE GG->CC mutant caused a similar disruption of protein–DNA interaction with the notable exception that at the lowest ratio of IRF-2 to ISRE DNA tested (17:0.3), some DNA remains unbound for both the ISRE WT and ISRE GG->CC DNA duplex (Figure 3C, lanes 9 versus 4). This suggests IRF-1 has a greater affinity for CEACAM1’s ISRE than IRF-2 and under natural conditions this difference could be important for modulating transcriptional activity at the promoter. Competition assays were performed with increasing amounts of competitor ISRE-WT DNA in the presence of 0.3 pmol ISRE GG->CC DNA and either 67 pmol IRF-1 or IRF-2 protein (Figure 3C). We observed competitor DNA caused accumulation of complex C2 in ISRE GG->CC DNA (see lanes 11 and 12 versus 5 and 6) which agrees with the predicted enhanced affinity of ISRE WT for both IRF-1 and IRF-2. Addition of 3.2 pmol competitor WT DNA, which produced all three binding states (unbound, yellow C2 and C1 complexes which is composite of red and green channel, lanes 6 and 12), indicates complete saturation of exogenous IRF protein. Taken together, these data validate the importance of the dinucleotide 223-GG-224 in the ISRE of CEACAM1.

### Splicing reporter constructs show production of L-isoform requires only IRF-1

**Figure 4:**
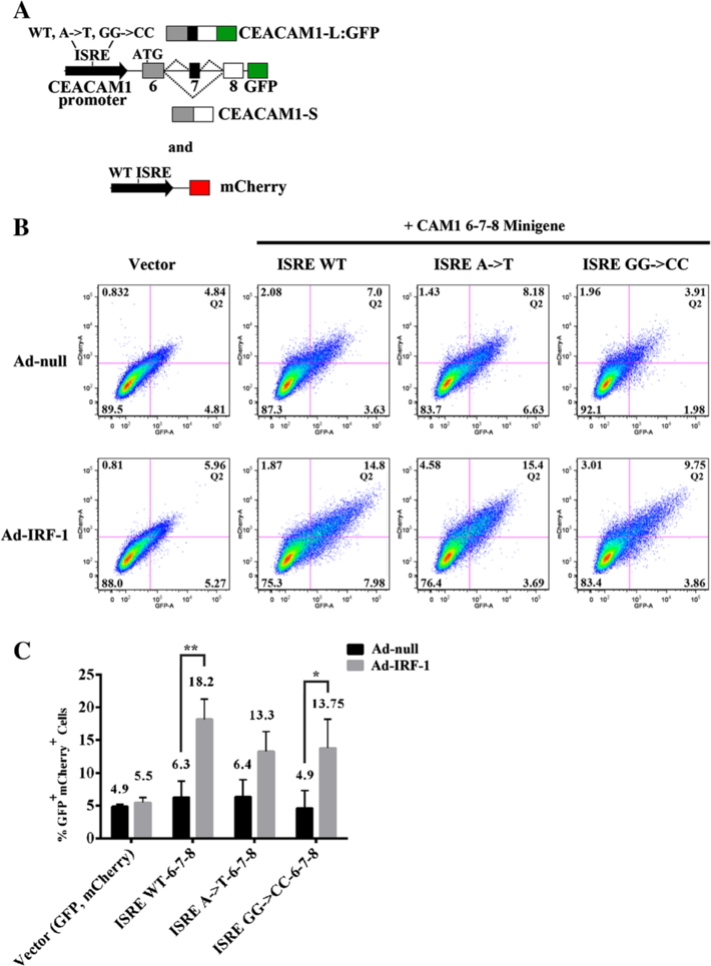
**Mutations in CEACAM1 promoter fail to ablate GFP expression when fused to CEACAM1 6-7-8 minigene. (A)** Schematic diagram of CEACAM1’s WT promoter and mutants A- > T and GG->CC (upper black arrow) were translationally fused to exons 6-7-8 minigene. Only AS to generate the L-isoform expresses the GFP reporter. A mCherry reporter that was co-transfected and used as a quantitation control was fused to CEACAM1 WT ISRE (lower black arrow). **(B-C)** Flow cytometric analyses, histogram and statistical analyses of Q2 in Figure 4B were similar to description in Figure 2 legend.

Having demonstrated GFP fluorescence correlates with induction of transcriptional regulation in our Ad-IRF-1 system, we next generated a tripartite splicing reporter construct consisting of a WT or mutant ISRE fused to the CAM1 6-7-8 minigene immediately upstream of the GFP reading frame such that fluorescence activity occurs from a single mRNA (Figure 4A). Essentially, these splicing reporters utilize AS mechanisms to modulate on–off expression of GFP such that exon inclusion but not exon skipping of a variable region puts GFP in frame. As shown in Figure 4B, one of three independent experiments, and summarized in Figure 4C, mock-treated ISRE WT samples but not vector controls produced double positive GFP/mCherry cells (6.3% compared to 4.9%). This correlated well with the fact that the default splicing program for CEACAM1 in MDA-MB-468 cells is the short isoform (see Figure 1C, lanes 2-3). Intriguingly, the percent double positive cells observed for the ISRE A->T mutant did not decrease significantly in comparison to the ISRE WT (6.4% compared to 6.3%) and the ISRE GG->CC mutant was now similar to background vector control levels (4.9% compared to 4.9%). In direct contrast to our promoter-only driven GFP reporters (Figure 2), the addition of exogenous IRF-1 strongly increased reporter activity by a potent 2.9-fold in the ISRE WT reporter [18.2/6.3; p < 0.01] as compared to the vector control that showed no significant difference. Flow cytometry of the ISRE GG->CC mutant revealed similar trends in its ability to stimulate GFP expression (Figure 4C; 13.75% compared to 4.9%; p < 0.05). From these data, we conclude that IRF-1 can activate spliceosome processing leading to the generation of CEACAM1-L. Notably, recovery of double positive GFP/mCherry cells in the ISRE A->T and ISRE GG->CC mutants to levels approaching the ISRE WT strongly indicated the possibility that regulatory elements were compensating for the strong differential potencies of these mutants.

### Exon 7 directs AS through promoter activation of CEACAM1

We next tested if the promoter itself was responsible for recruiting splicing factors, such as hnRNPs, to the site of transcription. Proteins important for initiation of the transcription machinery, such as Med23 of the Mediator Complex, have been shown to interact with hnRNP L in the recruitment function for the preinitiation complex formation of the spliceosome [35]. In previous studies, we have demonstrated that binding of hnRNP L and hnRNP A1 bind to exonic silencer sequences within exon 7 of CEACAM1 to repress exon inclusion [11]. Additionally, we further demonstrated an extensive spliceosome regulatory complex specifically assembles on exon 7, presumably containing a multiplicity of splicing factors. We therefore proceeded to test the hypothesis that exon 7 RNA acts as a regulatory element in the promoter activation of CEACAM1.

**Figure 5:**
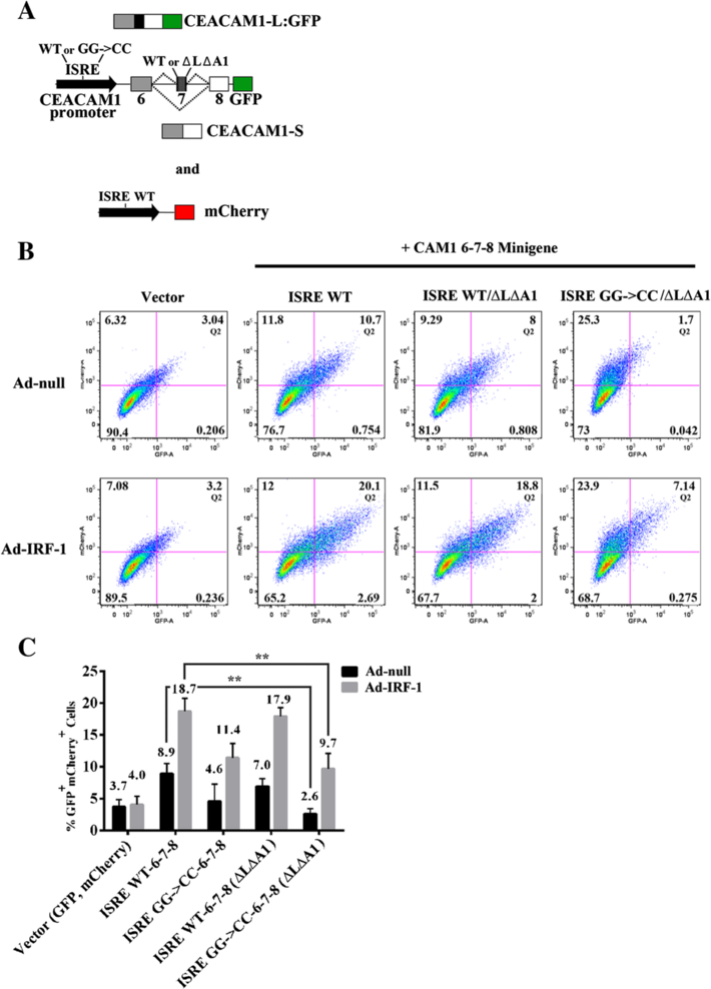
**Ad-IRF-1 is necessary and responsible for inducing AS in CEACAM1 to generate the L-isoform. (A)** Schematic diagram of CEACAM1’s promoter (WT; upper black arrow) and mutant GG- > CC translationally fused to exons 6-7-8 minigene. Only AS to generate the L-isoform expresses the GFP reporter. Mutations that ablate the hnRNP L and hnRNP A1 binding sites on exon 7 (black box) are indicated (ΔLΔA1). A mCherry reporter that was co-transfected and used as a quantitation control was fused to CEACAM1 WT ISRE (lower black arrow). **(B-C)** Flow cytometric analyses, histogram and statistical analyses of Q2 in Figure 5B were similar to description in Figure 2 legend.

Our experimental approach consisted of coupling ISRE promoter mutations with substitution of the cis-elements involved in hnRNP L and hnRNP A1 binding in exon 7 and studying the effects of splicing regulation on the production of CEACAM1-L:GFP. To effectively delete the binding sites of these splicing factors, we targeted two regions for further mutagenesis, namely E7-5 (nt 21-30) and E7-9 (nt 41-50) of exon 7 (total 53 nt). Previously we had shown potent exon-inclusion regulatory responses during our scanning mutagenesis study in the areas of exon 7 [see Figure 1B in [11]]. For simplicity, we refer to these RNA elements from this point forwards as ΔL (hnRNP L) and ΔA1 (hnRNP A1) (Figure 5A). As shown in Figure 5B, one of three independent experiments, and summarized in Figure 5C, mock-treated ISRE WT samples but not vector controls nor the ISRE GG->CC mutant produced double positive GFP/mCherry cells (8.9% compared to 3.7% and 4.6%). Consistent with our hypothesis of how hnRNP A1 and hnRNP L coordinate spliceosome functioning to generate the CEACAM1-S isoform, WT ISRE coupled-exon 7 ΔLΔA1 double mutants revert double positive GFP/mCherry cells towards WT levels (7.0% compared to 8.9%). Most importantly, the ISRE GG->CC-6-7-8 (ΔLΔA1) mutant creates a striking interaction that decreases double positive GFP/mCherry cells compared to WT ISRE-6-7-8 (2.6% compared to 8.9%; p < 0.01). When IRF-1 is reintroduced to these double mutant promoter-exon 7 cells, we observed an impressive three-fold recovery of double positive GFP/mCherry cells strongly suggesting mutations that disrupt proper folding of exon 7 RNA can be rescued by overexpression of IRF-1. Taken together, these data support our hypothesis that interactions between the spliceosome and transcriptosome occur through IRF-1 (9.7% compared to 2.6%; p < 0.01) and a disruption of either complex dramatically alters AS.

**Figure 6:**
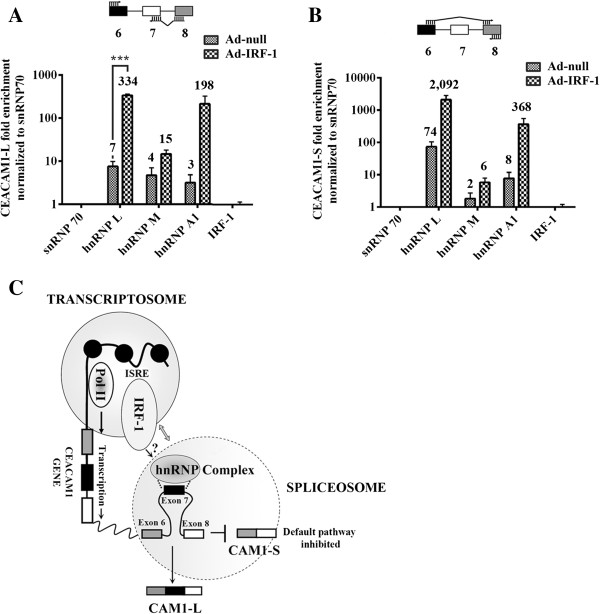
**RNP complexes containing hnRNP L, A1, and M associate with CEACAM1 mRNA. (A-B)** Quantitative RT-PCR of immunoprecipitated CEACAM1-L and CEACAM1-S mRNA. Primers were designed to either detect the presence or absence of exon 7, as shown above each figure. The average CEACAM1 mRNA fold enrichment is shown above each antibody (histogram) tested. **(C)** Model for the role of IRF-1 in coupling transcription and AS. In breast carcinoma epithelial cells default splicing maintains production of CEACAM1-S. When IRF-1 binds to its cognate ISRE sequence, the transcriptional complex communicates with the splicing machinery (denoted with <->) via uncharacterized interactions through splicing regulators leading to an AS switch and production of CEACAM1-L.

We next performed a series of RNA-protein binding experiments to probe whether the mechanism of IRF-1 associated CEACAM1 binding occurs through direct or indirect interactions. We hypothesized that slight changes in gene expression of hnRNP L and hnRNP A1 by IRF-1 might contribute to altered AS of CEACAM1. Ad-IRF-1 treated cells at 24 h were subjected to RIP assays, which can be used to identify specific RNA molecules associated with specific nuclear or cytoplasmic binding proteins in endogenously formed complexes. Using antibodies specific to hnRNP L, hnRNP M, hnRNP A1 and IRF-1, we tested for the ability to pull-down CEACAM1 transcripts that included or lacked exon 7 (Figure 6). We observed Ad-IRF-1 treatment led to a greater than 45-fold and 66-fold enrichment of CEACAM1-L mRNA by antibodies to hnRNP L and hnRNP A1, respectively versus Ad-null alone (Figure 6A). By contrast, we observed negligible association of CEACAM1-L mRNA by antibodies to hnRNP M. Similar trends were observed for CEACAM1-S mRNA, though more enrichment of short isoform mRNA was pulled down by antibodies to hnRNP L and hnRNP A1 (Figure 6B). This phenotype is reasonable given the translational induction of CEACAM1-S exceeds that compared to CEACAM1-L (see Figure 1D, lane 4 versus 3). hnRNP M did not strongly associate with CEACAM1 mRNA (Figures 6A-B), as expected since our RNA co-immunoprecipitation experiments never showed a direct interaction of hnRNP M with exon 7 RNA [11]. This supports the hypothesis that hnRNP M transiently associates with the spliceosomal complex that regulates exon 7 AS (Figure 1A). Taken together, these results also validate our previous finding that hnRNP A1 influences exon 7 despite not having a typical consensus sequence found in several eukaryotic genes suggesting that RNA species require interrogation beyond simple identification of local splicing factors and sequences [11]. Since we could not detect precipitation of CEACAM1 mRNA using IRF-1, we conclude that the effect of the CEACAM1 promoter on AS of CEACAM1 occurs through indirect interactions (Figure 6C).

### IRF-1 mediates Global AS

**Figure 7:**
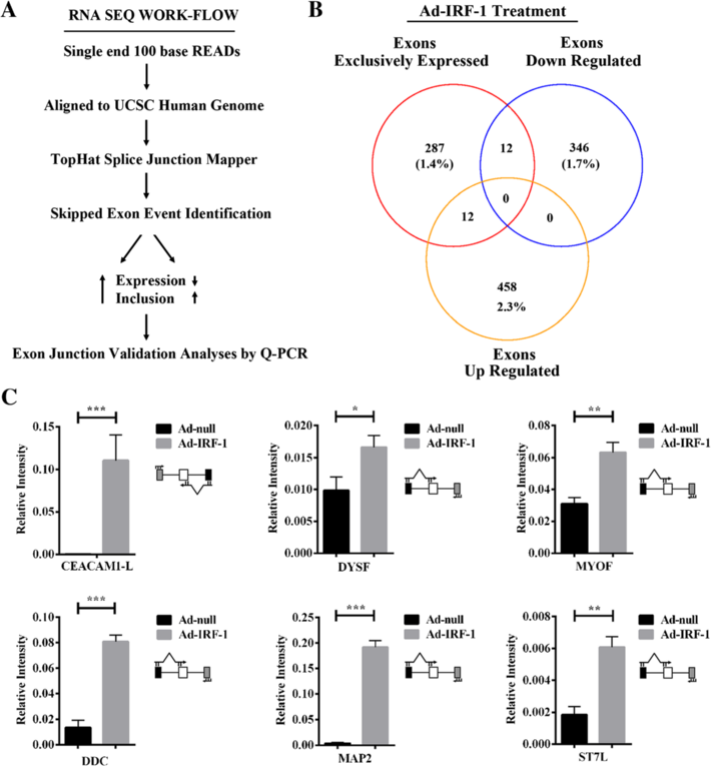
**RNA SEQ of Ad-IRF-1 treated breast carcinoma cells, MDA-MB-468. (A)** RNA SEQ Workflow. Reads were analyzed for exclusion, inclusion and differential gene expression in -/+ induction by Ad-IRF-1 after 24 h. Only genes with high inclusion but low expression reads were considered candidates for further study and validated by exon junction specific PCR. **(B)** A Venn diagram summarizing the overlap between genes differentially expressed. Exons exclusively expressed (representing exon inclusion; red circle), exons dowregulated (yellow circle) are shown in this array. **(C)** Quantitative RT-PCR of candidates as compared to control sample CEACAM1. Primer pairs were designed as exon junction to recognize upstream and variable exon with priming on the downstream exon as shown above the figure.

We next asked whether this ability of IRF-1 to participate in splice-site selection is representative of a generalized pathway of induction of molecular networks conferring putative host defense to the cell. We evaluated gene expression and AS levels from RNA-seq data following a workflow that was designed to identify skipped exon events in the transcriptome (Figure 7A, Additional file 5: Table S1). We evaluated our dataset for genes that were up (2.3%) or down-regulated (1.7%) and that had exons exclusively expressed (1.4%) in only the Ad-IRF-1 treatment (Figure 7B). When we focused our attention on genes that were either up-regulated with exons that were exclusively expressed or down-regulated with exons exclusively expressed, we observed subsets that included similar numbers of genes (12 each; Figure 7B). To provide evidence for the inclusion of the exclusively expressed exon, we performed quantitative reverse transcription PCR (using primer pairs as shown next to each gene studied; Figure 7C). The relative abundance of IRF-1-dependent transcripts were characterized for genes DYSF, MYOF, DDC, MAP2 and ST7L in comparison to housekeeping β-actin and a positive control of CEACAM1-L mRNA using isoform-specific primers (Figure 7C). We observed an approximately 5-fold (DDC; p < 0.001) to 20-fold (MAP2; p < 0.001) increase in the number of exons exclusively expressed in transcripts induced in IRF-1 treated samples as compared to the control Ad-null. Based on our customized bioinformatics approach and the small number of genes tested as a proof-in-principle, we predict many more genes may have their AS influenced by IRF-1 (Additional file 5: Table S1). We conclude that besides its role as a transcription factor, IRF-1 plays a significant role in the regulation of AS of human genes involved in a variety of cellular functions. This newly discovered role may augment the immune response by global changes in AS.

**Figure 8:**
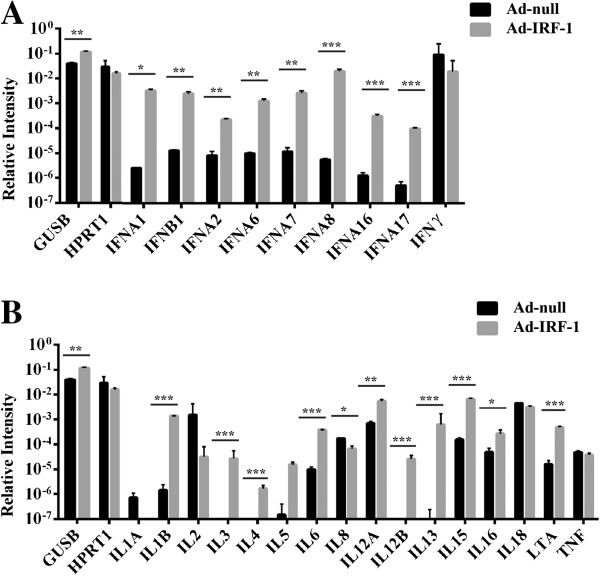
**IRF-1 can induce global splicing changes in the interleukin family of genes. (A)** Array of cytokine-network IFN-associated genes and endogenous control genes are indicated. **(B)** Interleukin-associated genes. Total RNAs from MDA-MB-468 cells were isolated, converted to cDNA and subjected to qRT-PCR. Data are expressed as ratio of cytokine gene expression to endogenous control gene signal intensities after culturing for 24 h. All assays are plated in triplicate. *, p < 0.05, **, p < 0.01; ***, p < 0.001 versus Ad-null control.

Since IRF-1 is regulated by IFNs, and since cytokine induction is associated with an increase in alternatively spliced mRNA transcripts [36,37], we next considered the possibility that cytokines that undergo AS are directly mediated by the expression of IRF-1. Using a human cytokine array, mRNA transcripts were analyzed by quantitative real-time PCR for cytokines representing Type I (IFN-α genes and IFN-β), Type II (IFN-γ), Type III (interleukins) and TNF superfamily (LTA) genes in comparison to housekeeping genes, GUSB and HPRT (Figure 8). We observed uniform and statistically significant transcriptional differences between untreated and treated Ad-IRF-1 infected cells for the Type I interferons, as expected. Most notably, this included IFNA1 (p < 0.05), IFNB1, IFNA2, IFNA6, IFNA7 (all p < 0.01) and IFNA8, IFNA16, IFNA17 (all p < 0.001). We also observed highly responsive changes in expression in the interleukins, most notably IL1β, IL3, IL4, IL12B, IL13 and IL15 which showed several orders of magnitude difference (all p < 0.001). Taken together, these data extend the number of genes that are under the control of AS master regulator, IRF-1.

**Figure 9:**
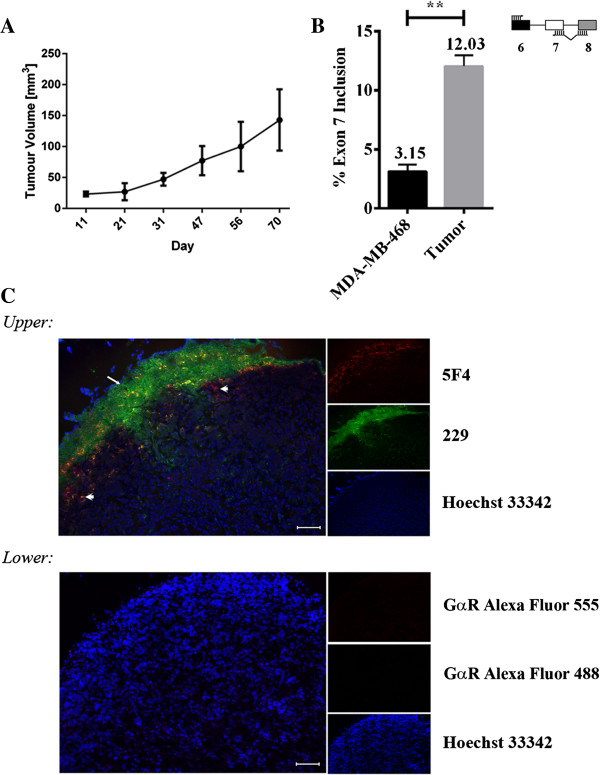
**Expression pattern of cytoplasmic isoforms of CEACAM1 *in vivo*. (A)** Mean MDA-MB-468 tumor volumes (±SD) orthotopically implanted into mammary fat pads of NOD/SCID mice (n = 6). **(B)** Total RNAs were isolated from 6 tumors and two parental cell populations. RNAs were subjected to quantitative PCR using exon-junction specific primers. The % exon 7 inclusion was calculated by taking the CEACAM1-L/S ratio in tumor cells and comparing this fold increase to the CEACAM1-L/S ratio in parental cells. **(C)** Immunofluorescence staining of human CEACAM1 expressing cells from MDA-MB-468 tumor tissue. (*Upper):* Expression of CEACAM1-L but not CEACAM1-S is more intense at the invasive front of the breast carcinoma tissue. Immunofluorescence staining of human CEACAM1 expressing cells from MDA-MB-468 tumor tissue using antibodies directed to the ectodomain shown in red (5F4 mouse antibody 1:200; shown with white arrowheads) or the -L cytoplasmic tail of CEACAM1 shown in green (229 rabbit antibody 1:200; shown with white arrow) followed by secondary antibodies (goat anti-rabbit, Alexa Fluor 488 labeled and goat anti-mouse, Alexa Fluor 555 labeled; 1:200). For nuclei staining, Hoechst 33342 (blue) was used at final concentration of 1 μg/ml. Scale bar, 100 μm. (*Lower):* Secondary antibodies only were used as negative controls to rule out interference from auto-fluorescence.

Since the CEACAM1 long isoform contains an ITIM, a characteristic motif found in immune cells, we next addressed the potential impact of the isoform switch in the connection to tumorgenesis where an imflammatory environment is considered pro-tumorigenic. For this study, we chose to continue with our model breast cancer cell line MDA-MB-468 and asked how the CEACAM1 isoform ratio would behave in a tumor. MDA-MB-468 cells were orthotopically implanted into mammary fat pads of NOD/SCID mice (n = 6) and tumor volumes were measured until they reached an average size of 150 mm^3^ (Figure 9A). Total RNAs were isolated from 6 tumors and two parental cell populations and subjected to quantitative PCR using human exon-junction specific primers for CEACAM1. Our data shows that the cytoplasmic domain of CEACAM1, produced primarily as a short isoform in the parental cells grown on plastic, undergoes a significant 4-fold induction (12.03/3.15) of AS to generate the cytoplasmic L-isoform (Figure 9B). Immunofluorescence staining of human CEACAM1 expressing cells from MDA-MB-468 tumor tissue using antibodies directed to the ectodomain shown in red (5F4) or the -L cytoplasmic tail of CEACAM1 (229) shown in green, showed strong expression along the invasive front of the breast tumor (Figure 9C; *Upper*) as compared to control secondary antibodies (Figure 9C; *Lower*). When we investigated whether invasive CEACAM1 positive mouse cells on the tumor front might be responsible for the AS we observed for human CEACAM1 MDA-MB-468 cells (Additional file 6: Figure S5), we observed little staining with antibody CC1, which recognizes mouse CEACAM1 exclusively. Taken together, our results with human CEACAM1 are consistent with recent findings that showed increased expression of the L-isoform associates with the invasive front in colorectal cancer [38] and that the S/L isoform ratio of CEACAM1 is altered in breast cancer specimens [17]. We conclude that differences between long and short cytoplasmic isoforms occur in human breast tumor CEACAM1 expressing cells, and are influenced by the tumor microenvironment, and specifically, favor the long isoform characteristic of immune cells rather than the normal epithelial short isoform.

## Discussion

### CEACAM1 AS is dependent upon IRF-1 activity

In this study, we have shown that promoter regulation by IRF-1 determines CEACAM1 AS, specifically to generate the cytoplasmic L-isoform. Previously we proposed that regulatory cis-acting elements in exon 7 in CEACAM1 in combination with the relative abundance of splicing factors hnRNP L, hnRNP A1, and hnRNP M play the critical role in the AS of CEACAM1 (Figure 1; [11]). Here we extend our findings to include functional interactions between the promoter of CEACAM1 and exon 7 RNA that play a role in isoform-specific splicing.

The critical observation that led to this study was that type II IFN-γ led to the AS of CEACAM1 in breast carcinoma cells, MDA-MB-468 [8]. Previously, it was reported that IFN-γ can cause the AS of tryptophanyl-tRNA synthetase, though the direct mechanism causing this splicing switch was not explored [39]. IFN-γ is an essential mediator of the immune response to viruses and cancer and is one of the strongest inducers of IRF-1 expression in cancer cells. While IRF-1 is itself subject to AS [40,41], this is the first study to show that IRF-1 coordinates promoter activity and AS to control global changes in the cell. Previous studies have shown that promoter identity influences alternative RNA-processing decisions, through kinetic Pol II elongation and chromatin structure [42]. For example, the catalytic subunit Brm of the SWI/SNF chromatin-remodeling complex was shown to favor inclusion of variant exons in the mRNA of several genes, including E-cadherin, BIM, cyclin D1 and CD44 [43]. CoAA, an hnRNP-like protein, was shown to mediate transcriptional and splicing effects through interactions with transcriptional coregulator TRBP, a protein recruited to target promoters through interactions with activated nuclear receptors [44]. From the point of view of the spliceosome, there are few studies that show how splicing factors mechanistically control transcription.

The novelty of this work lies in the following. First, we show that IRF-1 can control AS of CEACAM1 and a number of other genes involved in cellular maintenance and differentiation (Figures 1, 7, 8). Second, CAM1 6-7-8 minigenes were genetically fused to CEACAM1 promoter and GFP, where only the L-isoform indicates an authentic AS event. Third, to prove that pre-mRNA processing events were temporally coupled to IRF-1, we show that mutations in the ISRE that caused a loss-of-function phenotype in promoter-only studies (Figure 2) in Ad-null/MDA-MB-468 transfected cells were no longer effective (Figure 4; see explanation to follow). When Ad-IRF-1/MDA-MB-468 transfected cells were analyzed, we observed 3-fold increases in GFP expression in our mutant constructs strongly validating a role for IRF-1 in the AS of CEACAM1. Fourth, to prove that compensation of promoter activity was coming from the CEACAM1 minigene RNA, we created double mutations in the promoter and exon 7 RNA and showed pre-mRNA processing events were temporally coupled (Figure 5). Indeed, we show that cellular-driven AS fates in CEACAM1 expression of the L-cytoplasmic tail can secondarily co-regulate its own gene transcription, in a mechanism similar to how *Drosophila melanogaster* embryonic transcription units show intron looping and associated ribonucleoprotein complexes on transcripts tethered to DNA [45,46] (Figure 6C). Our data suggests that in breast carcinoma epithelial cells the default splicing maintains production of CEACAM1-S. When IRF-1 binds to its cognate ISRE sequence, the transcriptional complex communicates with the splicing machinery via uncharacterized interactions through splicing regulators leading to an AS switch and production of CEACAM1-L. Fifth, we infer that SP1 and USF1, known transcription factors associated with the CEACAM1 promoter [8], do not rescue loss-of-function mutations in Ad-null/MDA-MB-468 transfected cells with our CEACAM1 ISRE:CAM1 6-7-8 reporter constructs (Figures 2, 4-5) strongly suggesting that IRF-1 is unique among the transcription factors for generating the CEACAM1 long ITIM containing isoform.

### A Global role for IRF-1 and AS

While global characterization of IRF-1 genes in vertebrates as a mechanism for host-defense has been studied extensively [47], the study of how IFN-γ via IRF-1 increases the complexity and diversity of the immune system through AS is rather limited. Host defense serves two main functions: the generation of immune responses to invading pathogens and the suppression of tumor development. These are achieved by the efficient coordination of prompt and controlled cellular responses. Additionally, tight control of AS can be influenced by external conditions (e.g., the circadian clock [48], nutritional state [49], and cell-cycle controls [50], for example) to function as specific regulators of gene expression. In this respect, our work adds to the growing body of evidence that links AS to environmental cues that activate the spliceosome, in this case immune activation through IRF-1. Our RNA-sequencing and cytokine studies (Figures 7, 8) provide new evidence for a function beyond the known IRF-1 effector responses by showing that multiple genes involved in a wide-array of cellular functions are subject to IRF-1 AS (Figure 7; Additional file 5: Table S1).

### *In vivo* generation of an ITIM-containing isoform

Breast cancers are phenotypically and genotypically heterogeneous tumors containing multiple cancer cell populations with various metastatic potential. In normal breast epithelial cells, where CEACAM1 is predominantly expressed as the S-isoform, but the S/L ratio is altered in breast cancer [17], one can ask if this change is a consequence of the inflammatory tumor microenvironment, and furthermore, if this change is advantageous to tumor growth and/or invasion. First, it is noteworthy that immune cells express the long and not the short isoform, perhaps due to their own response to inflammatory signals that include IFN-γ produced by activated T- and NK cells [51]. Second, the change in microenvironment during tumorigenesis may cause this change in AS, again via IFN-γ and IRF-1. This phenotype has been reported previously in colon cancer, where the long cytoplasmic dominance of CEACAM1 was associated with invasion and migration of colorectal cancer cells [38]. Indeed, in our study we demonstrate that a similar AS change of CEACAM1 was observed at the invasion front of breast cancer cells (Figure 9). This finding is consistent with recent studies that delineate the role of the microenvironment in determining tissue-specific CEACAM1 S or L dominance in immune cells [52] and in creating a pro-angiogenic tumor microenvironment [53]. Finally, it is worth considering if expression of an inhibitory ITIM containing isoform is pro-tumorigenic. The answer to this question will require further delineation of the signaling pathways in these cells, a complex issue to say the least.

## Conclusions

Taken together, our results reveal a novel role for IRF-1 in the cotranscriptional processing of RNA. In this study, we have shown that promoter regulation by IRF-1 determines CEACAM1 AS, specifically to generate the cytoplasmic L-isoform. Effector responses by IRF-1 in the context of AS should reveal novel cellular functions. Our work is consistent with the idea that immune cells reacting to invading areas of a growing tumor generate IRF-1 in the tumor perhaps through release of IFN-γ, which eventually leads to AS to generate CEACAM1 L-isoform expression not only on T cells but in non-inflamed epithelial cells as well. In cases of acute infection this sort of temporary change in phenotype may be advantageous, while a chronic pro-inflammatory phenotype associated with tumor growth may favor further tumor growth.

## Methods

### Cell culture, reagents, and treatments

MDA­MB­468 (ATCC® HTB­132™) and MCF7 (ATCC® HTB-22) cells were purchased from ATCC and grown in a 5% CO_2_ incubator at 37°C in MEM supplemented with 1% Sodium Pyruvate, 1.5% Sodium Bicarbonate, 1 × Non-essential Amino Acids, 1 × Penicillin/Streptomycin/Amphotericin B and 10% heat-inactivated FBS. For induction of IRF-1, mouse IRF-1 was used essentially as described previously except for the following modifications [29]. Cells were seeded at a density of 0.5 × 10^6^ cells in 6-well plates 24 h prior to treatment. Recombinant mouse IRF-1 adenovirus (hereafter referred to as Ad-IRF-1) or the Ad-null empty vector control was added to cultures at PFU estimated at 100 particles per PFU. Cells were incubated for 24 h at 37°C and 5% CO_2_ unless otherwise specified. After incubation, RNA and proteins were isolated as described previously [11]. For Western blots, FACS and immunofluorescent staining identifying CEACAM1 isoforms anti-CEACAM1-L (229; [54]), T84.1 and 5F4 were used. For CEACAM1 specific antibody, 5F4 was a kind gift of R. Blumberg (Harvard University). Antibodies used for Western blots identifying IRF-1 (ab26109), IRF-2 (C-19, sc-498), GAPDH (FL-335, sc-25778) and p21 (EA10, ab16767) were purchased from Santa Cruz Biotechnology or from Abcam.

### RNA isolation semi and quantitative RT-PCR

Total RNA was isolated by the RNeasy mini kit (Qiagen). The RNA was treated with RNase-free DNase set (Qiagen), and RNA (1 μg) was reverse-transcribed in a 20 μl reaction using Maxima First Strand cDNA Synthesis Kit for RT-PCR (K1641, Thermo Scientific) following manufacturer instructions. Reactions were diluted 5-fold and 1/50 of cDNA were used for semiquantitative PCR with gene-specific primers and 2x Taq PCR Premix (Bioland Scientific) for 30 cycles for standard RT-PCR. Primers designed to amplify CEACAM1-3 and CEACAM1-4 isoform were described previously [7]. Amplification of CEACAM1 mRNA for RT-PCR was performed by using sense exon 6 primers (5’-TTCTGCATTTCGGGAAGACCGGCAG-3’) coupled with antisense 3’ UTR primers (5’-AGCCTGGAGATGCCTATTAG-3’). Primer amplification regions for GFP are indicated in Figure 2C. The products were resolved on 2.25% agarose gels and visualized by staining with SYBR Green I (Invitrogen). Gels were photographed on a GelLogic 200 Imaging System. For real time PCR, 1/50 of cDNA reverse transcription reactions were used to amplify constitutively expressed housekeeping gene β-Actin and CEACAM1 mRNA levels. The reactions were performed on a CFX96 Touch Real-Time PCR Detection System (Bio-Rad) in a 20 μl volume with SsoFast EvaGreen Supermix (Bio-Rad). Primers BP56 (5’- GGTTGCTCTGATAGCAGTAGCCCTGGCATGTTTTCTGCATTTCGGGAAGACCGGCAGCTC-3’) and BP60 with a common sense primer to exon 6 amplify differentially spliced isoforms, CEACAM1-S and CEACAM1-L, respectively [55]. TaqMan Array Human Cytokine Network (Life Technologies) plates were carried out according to manufacturer instructions. After denaturation for 30 sec at 95°C, 40 cycles were performed (5 sec at 95°C, 5 sec at 60°C). At the end of the reaction, melting curves were generated between 65°C and 95°C, for every 0.5°C. CEACAM1 mRNA and genes identified in our RNA Seq study were calculated by using β-Actin for normalisation. Quantification of gene expression was calculated by using the ΔCT method [56].

### Promoter and splicing reporters

All DNA constructs were generated by standard cloning procedures and confirmed by sequencing. Construction of promoter CEACAM1 WT ISRE:GFP plasmid proceeded by amplifying a genomic DNA fragment corresponding to -1,135 bp relative to the transcription start site (TSS) from MDA-MB-468 cells using *Bgl*II and *Hnd*III restriction sites cloned into pZsGreen-1 (Clontech). To construct CEACAM1 WT ISRE:CAM1 6-7-8:GFP plasmid, the *Bam*HI restriction site in the CEACAM1 promoter was disrupted to allow ligation of our minigene splicing cassette containing genomic CEACAM1 exons 6 through exon 8, described previously [11], using *Sac*II and *Bam*HI restriction sites. AS events that favored only L-isoform led to the excitation of GFP fluorescence. To control for external validity and allow for quantitation of transfection efficiency, promoter and splicing reporter experiments were conducted using a second co-transfected promoter CEACAM1 WT ISRE:mCherry plasmid. Mutations for all constructs were introduced using QuikChange Lightning Site-Directed Mutagenesis Kit (Agilent Technologies) following manufacturer’s instructions. To delete both hnRNP L and hnRNP A1 binding sites in exon 7, the sequence AAGCATGCAA was introduced using GeneArt mutagenesis (Life Technologies). This sequence does not associate with any known cellular factor and therefore does not influence AS [57].

### Fluorescence-based electrophoretic mobility shift assay (fEMSA)

The fEMSA was operated using a Light Shift Chemiluminescent EMSA kit (Thermo Scientific) according to the manufacturer’s instructions with the only exception that IRDye fluorescence-labeled oligonucleotides for ISRE WT or ISRE GG->CC were used to visualize DNA-protein binding interactions as described previously [58]. EBNA was used as a positive control for the assay reagents. Sequence information for each set of oligos is contained in Figure 3A. The DNA-protein binding reaction buffer was composed of 1X binding buffer supplemented with 5% Glycerol, 10 mM MgCl_2_, 200 ng Poly (dI-dC) final concentration and 0.3 pmol IRDye-labeled oligonucleotides in a total volume of 10 μl. Recombinant IRF-1 and IRF-2 (ProSpec) each containing a His-tag were used in binding reactions and the reactions were incubated at room temperature for 30 min before separation of complexes on a 6% DNA Retardation Gel (Invitrogen) at 100 V for 60 min. Detection of DNA binding was performed using Licor Odyssey infrared scanning using the following parameters: 700 and 800 nm channels, focus height 3.5 mm, and intensity set to 5.

### Flow cytometric analysis

Promoter and splicing reporters were transfected using cells at a density of 1 × 10^6^ cells using Amaxa Cell Line Nucleofector Kit V (Lonza) and 6 μg DNA from each construct. To pre-heated 6-well plates containing growth media, cells were electroporated and immediately incubated for 24 h before flow cytometric analyses using a BD Fortessa (Becton Dickinson, San Jose, CA). GFP and mCherry fluorescence intensities were detected with 488 nm and 561 nm lasers, respectively. Data were analyzed with FlowJo Software version 8.1.1 (Treestar).

### RNA immunoprecipitation

We performed RNA immunoprecipitation (RIP) experiments using the Magna RIP RNA-Binding Protein Immunoprecipitation Kit (Millipore, Bedford, MA), according to the manufacturer’s instructions. Antibodies to hnRNP L (4D11, sc-32317), hnRNP A1 (4B10, sc-32301), and hnRNP M (3C181, sc-56702) were purchased from Santa Cruz Biotechnology. The co-precipitated RNAs were detected by RT-PCR. Total RNAs (input controls) and isotype controls were assayed simultaneously to demonstrate that the detected signals were from RNAs specifically binding to CEACAM1 mRNA (data not shown). snRNP 70 antibody was used as a negative control for CEACAM1 binding.

### Bioinformatics

Sequences obtained from Illumina’s CASAVA pipeline were aligned to human genome assembly hg19 using Tophat v1.2.0 [59]. The expression levels of Refseq transcripts were calculated by the number of reads falling into the exon region of each transcript. The expression data were then quantile normalized and log2 transformed. Differentially expressed transcripts were identified by the following criteria: 1) Chi-square p value < 0.05; 2) Log2 ratio > 1 or < -1; 3) at least one sample has coverage > 10, where the coverage was calculated by the expression level of the transcript divided by the transcript length. The up-regulated and down-regulated genes were then subject to functional annotation analysis with DAVID (http://david.abcc.ncifcrf.gov/). A customized bioinformatics pipeline was developed to detect exon skipping event in the transcriptome. Briefly, for each Refseq transcript with 3 or more exons, the middle exons excluding the first and last exon were kept for further analysis. When exons were shared by multiple transcripts, only one exon was kept. We defined inclusion reads and exclusion reads as described previously [60]. The inclusion and exclusion reads were counted in both control and IRF-1 treated samples. The inclusion/exclusion ratio for each sample was then calculated. The potential skipped exons in control sample were identified by the following criteria: 1) inclusion reads in control = 0; 2) exclusion reads in control > 1; 3) inclusion reads in Ad-null > =5; 4) exclusion reads in Ad-null =0; 5) Difference of the inclusion/exclusion ratio Ad-null vs. control sample > 8-fold. Similar approach was used to identify skipped exons in the Ad-IRF-1 sample. These combinations of criteria were used to reduce the chance of false positives. The identified exons were then annotated back to Refseq transcripts and compared to differentially expressed transcripts identified above.

### Tumor xenografts in NOD/SCID mice

NOD/SCID transgenic mice were purchased from the Jackson Laboratory. MDA-MB-468 tumor cells (1 × 10^6^) were mixed with Matrigel (BD Biosciences) and orthotopically injected into mammary fat pads. The experiment was terminated after 70 days when tumors reached an average size of 150 mm^3^. Mouse care and experimental procedures were performed under pathogen-free conditions in accordance with established institutional guidance and approved protocols from the Institutional Animal Care and Use Committee of the Beckman Research Institute of City of Hope.

### Immunofluorescent staining

Frozen tumor tissue sections were fixated in 2% paraformaldehyde and permeabilized in methanol. After blocking first using Mouse on Mouse (M.O.M.) Blocking Reagent (Vector Lab) and then in PBS containing 10% goat serum for 1 h each, slides were incubated overnight with antibodies: mouse anti-human CEACAM1 ectodomain (5F4, 1:200), rabbit anti-human -L cytoplasmic tail of CEACAM1 (229, 1:200) [54] or mouse anti-mouse CC1, a kind gift of K. Holmes (University of Colorado). The following day the slides were washed and secondary antibodies were applied for 1 h (goat anti-rabbit, Alexa Fluor 488 labeled and goat anti-mouse, Alexa Fluor 555, both 1:200, Invitrogen) were visualized using an Olympus IX81 Inverted microscope with Q Imaging Retiga 2000R cooled CCD camera with Image Pro Plus version 6.3 imaging software and 40X magnification. For nuclei staining, Hoechst 33342 (blue) was used at final concentration of 1 μg/ml.

## Competing interest

The authors declare that they have no competing financial interests.

## Authors’ contributions

Experiments, study design and data analysis were performed by KJD. Animal studies were performed by MK. RIP and splicing assays were performed by DG. XW performed bioinformatics analyses. TN performed western blot and cytokine array analyses. CC helped with transient transfection experiments for reporter assays. JHY provided Ad-IRF-1. The manuscript was written by KJD and edited by JES. All authors read and approved the final manuscript.

## Supplementary Material

Additional file 1: Figure S1 Time-course of MDA-MB-468 breast carcinoma cells after induction with Ad-IRF-1. **(A)** Western blotting of cell lysates after no treatment and induction of Ad-IRF-1 after 6, 12 and 24 h with antibodies that recognize IRF-1 and GAPDH. **(B)** Analyses of mRNA expression by RT-PCR (*Upper*) and histogram representing the quantification of CEACAM1-L/S ratio as determined in Figure 1 (*Lower*). All assays were performed in triplicate. *, p < 0.05; ***, p < 0.001 versus Ad-null control. Click here for file

Additional file 2: Figure S2 Time-course of MCF7 breast carcinoma cells after induction with Ad-IRF-1. Analyses of CEACAM1–S **(A)** and CEACAM1–L **(B)** mRNA expression by qRT-PCR and histogram representing the quantification of CEACAM1 relative to β-Actin mRNA levels. All assays were performed in triplicate. **, p < 0.01; ***, p < 0.001 versus Ad-null control. Click here for file

Additional file 3: Figure S3 Western blotting of cell lysates after induction of Ad-IRF-1 after 24 h. Shown are fluorescent (700 nm and 800 nm) images probed with antibody 229 **(A)** and antibody T84.1 **(B)** to detect CEACAM1 isoforms (companion to composite figure shown in Figure 1D). Click here for file

Additional file 4: Figure S4 Flow-cytometry analyses of MDA-MB-468 breast carcinoma cells after induction with Ad-IRF-1. Antibody 5F4, which recognizes both isoforms of CEACAM1, was added to cells that were either mock treated or induced with Ad-IRF-1 after 24 h. Treatments are: Blank, cells not treated with antibody; 2^nd^ only, cells treated with Alexa Fluor 488 goat anti-mouse antibody only; Isotype, cells treated with mouse clone MG1-45 mIgG1 isotype control; MDA-MB-468, cells not treated with virus stimulation; Ad-null, cells treated with virus vector control; Ad-IRF-1, cells treated with virus expressing IRF-1. Click here for file

Additional file 5: Table S1 List of genes differentially expressed in the transcriptome analyses after IRF-1 treatment. Click here for file

Additional file 6: Figure S5 Expression pattern of mouse CEACAM1 *in vivo*. Immunofluorescence staining of mouse CEACAM1 expressing cells from MDA-MB-468 tumor tissue. CEACAM1 expressing cells from MDA-MB-468 tumor tissue were treated with antibodies directed to the ectodomain shown in red (CC1, mouse antibody 1:200; shown with white arrowheads) or the -L cytoplasmic tail of human CEACAM1 by comparison and shown in green (229 rabbit antibody 1:200; shown with white arrows) followed by secondary antibodies (goat anti-rabbit, Alexa Fluor 488 labeled and goat anti-mouse, Alexa Fluor 555 labeled; 1:200). For nuclei staining, Hoechst 33342 (blue) was used at final concentration of 1 μg/ml. Scale bar, 100 μm. Click here for file
