# Supplementary figures and images for: IRF-1 regulates alternative mRNA splicing of carcinoembryonic antigen-related cell adhesion molecule 1 (CEACAM1) in breast epithelial cells generating an immunoreceptor tyrosine-based inhibition motif (ITIM) containing isoform
Source: Mol Cancer. 2014 Mar 21;13:64. doi: 10.1186/1476-4598-13-64 (PMC4113144; doi:10.1186/1476-4598-13-64)

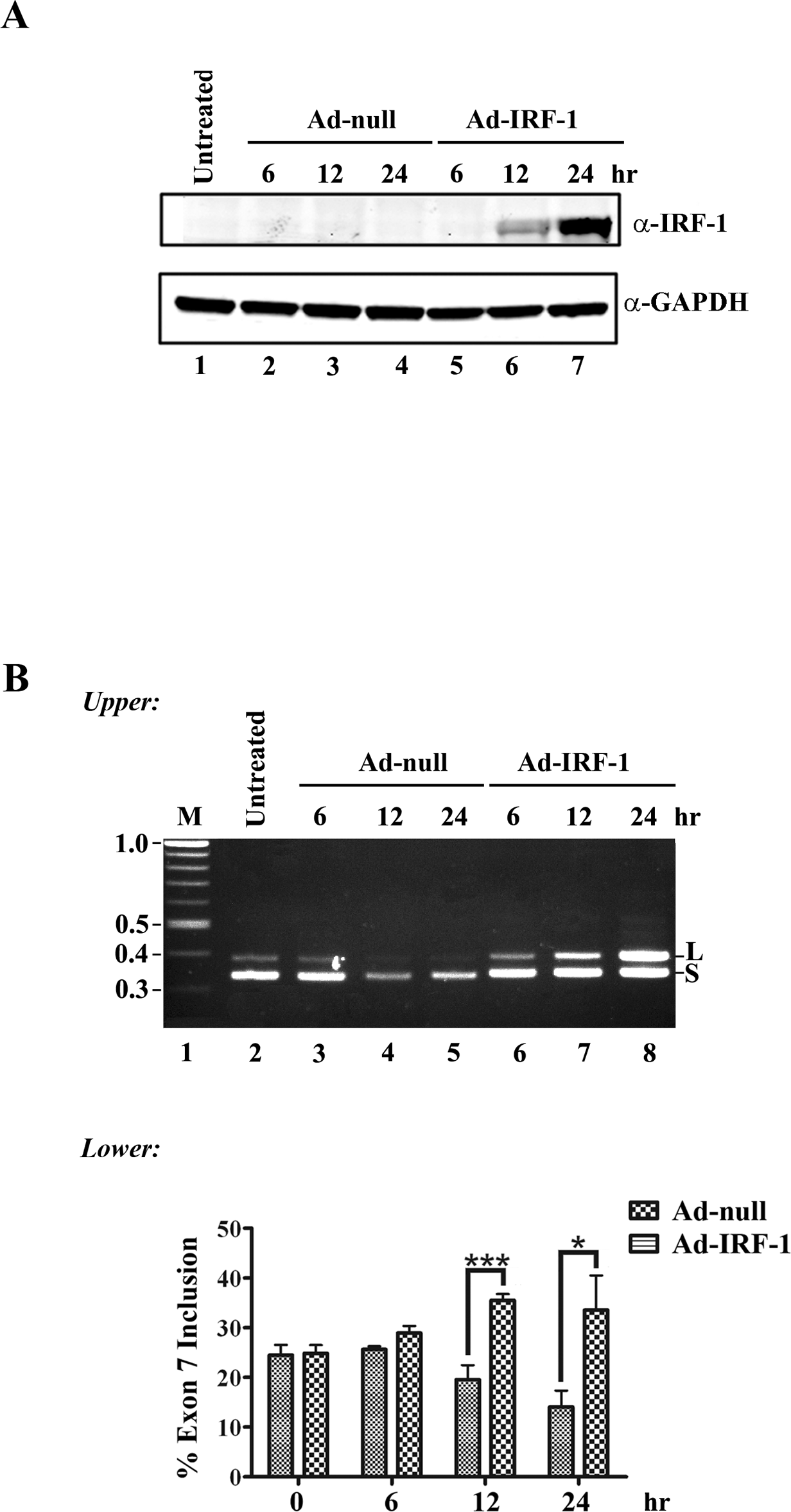

Supplement: Additional file 1: Figure S1 — Time-course of MDA-MB-468 breast carcinoma cells after induction with Ad-IRF-1. (A) Western blotting of cell lysates after no treatment and induction of Ad-IRF-1 after 6, 12 and 24 h with antibodies that recognize IRF-1 and GAPDH. (B) Analyses of mRNA expression by RT-PCR (Upper) and histogram representing the quantification of CEACAM1-L/S ratio as determined in Figure 1 (Lower). All assays were performed in triplicate. *, p < 0.05; ***, p < 0.001 versus Ad-null control. [file 1476-4598-13-64-S1.tiff]

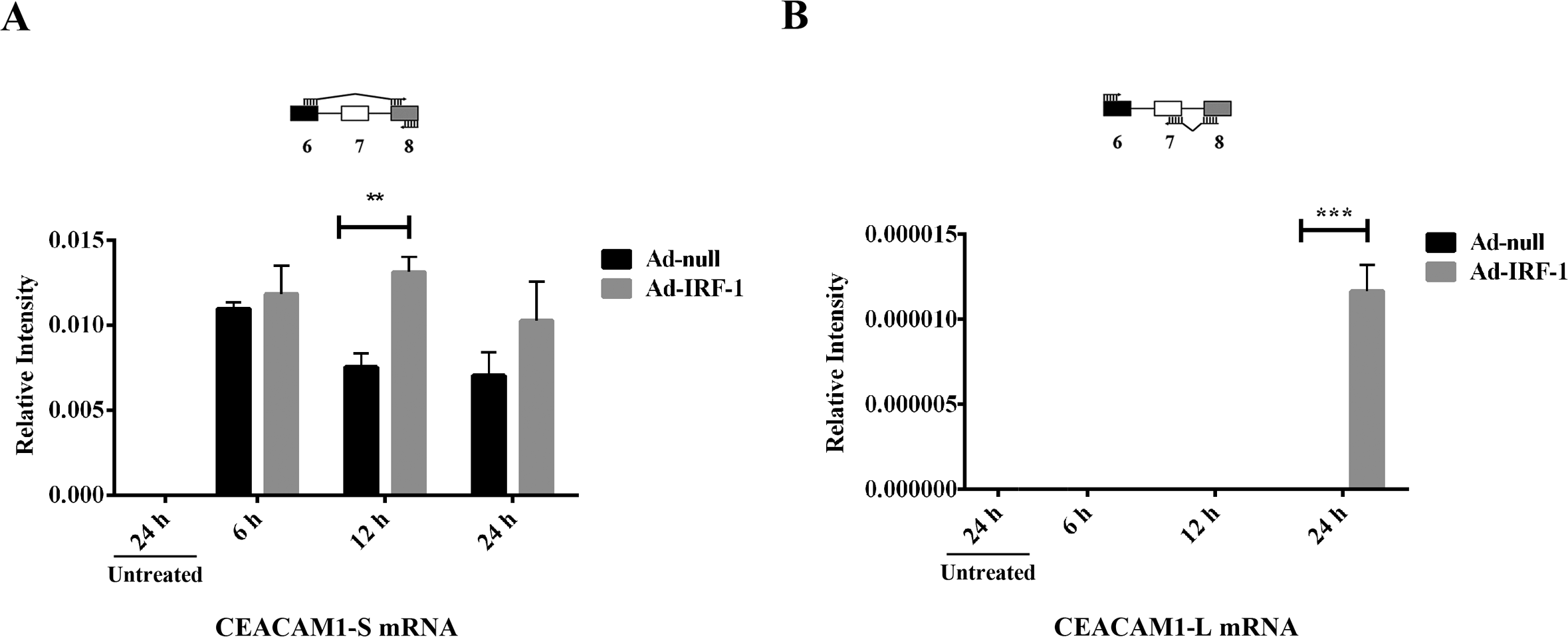

Supplement: Additional file 2: Figure S2 — Time-course of MCF7 breast carcinoma cells after induction with Ad-IRF-1. Analyses of CEACAM1–S (A) and CEACAM1–L (B) mRNA expression by qRT-PCR and histogram representing the quantification of CEACAM1 relative to β-Actin mRNA levels. All assays were performed in triplicate. **, p < 0.01; ***, p < 0.001 versus Ad-null control. [file 1476-4598-13-64-S2.tiff]

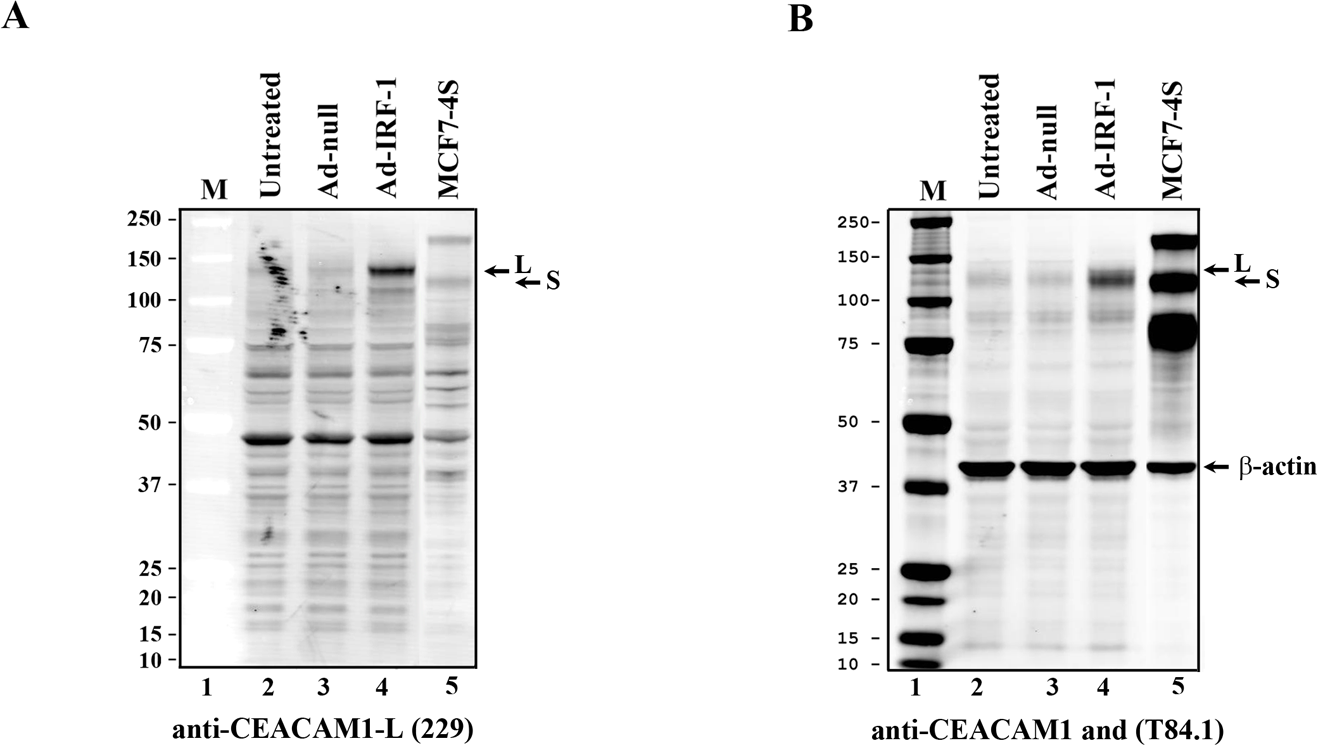

Supplement: Additional file 3: Figure S3 — Western blotting of cell lysates after induction of Ad-IRF-1 after 24 h. Shown are fluorescent (700 nm and 800 nm) images probed with antibody 229 (A) and antibody T84.1 (B) to detect CEACAM1 isoforms (companion to composite figure shown in Figure 1D). [file 1476-4598-13-64-S3.tiff]

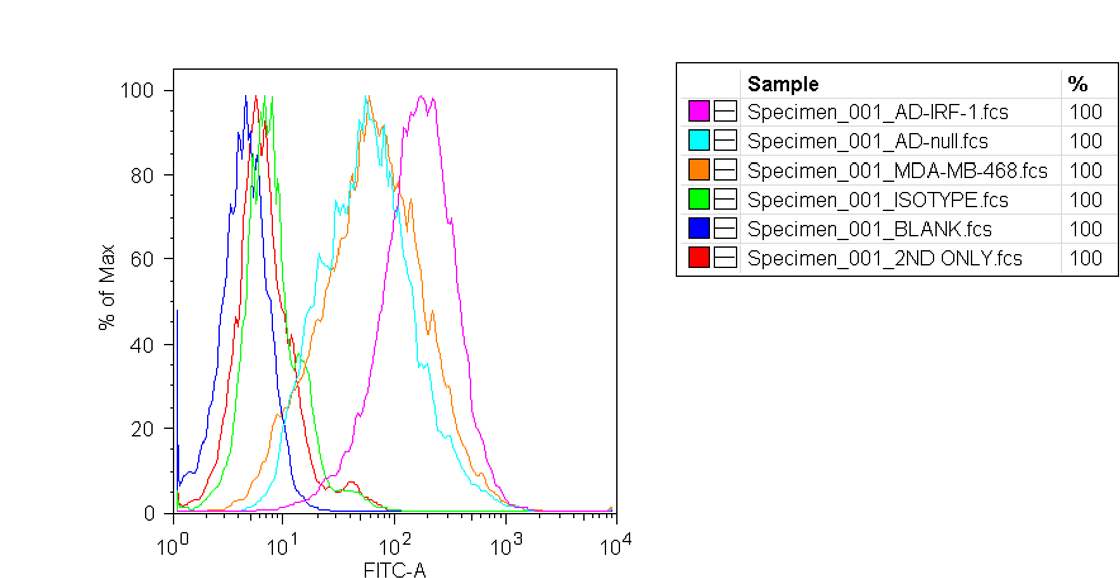

Supplement: Additional file 4: Figure S4 — Flow-cytometry analyses of MDA-MB-468 breast carcinoma cells after induction with Ad-IRF-1. Antibody 5F4, which recognizes both isoforms of CEACAM1, was added to cells that were either mock treated or induced with Ad-IRF-1 after 24 h. Treatments are: Blank, cells not treated with antibody; 2nd only, cells treated with Alexa Fluor 488 goat anti-mouse antibody only; Isotype, cells treated with mouse clone MG1-45 mIgG1 isotype control; MDA-MB-468, cells not treated with virus stimulation; Ad-null, cells treated with virus vector control; Ad-IRF-1, cells treated with virus expressing IRF-1. [file 1476-4598-13-64-S4.tiff]

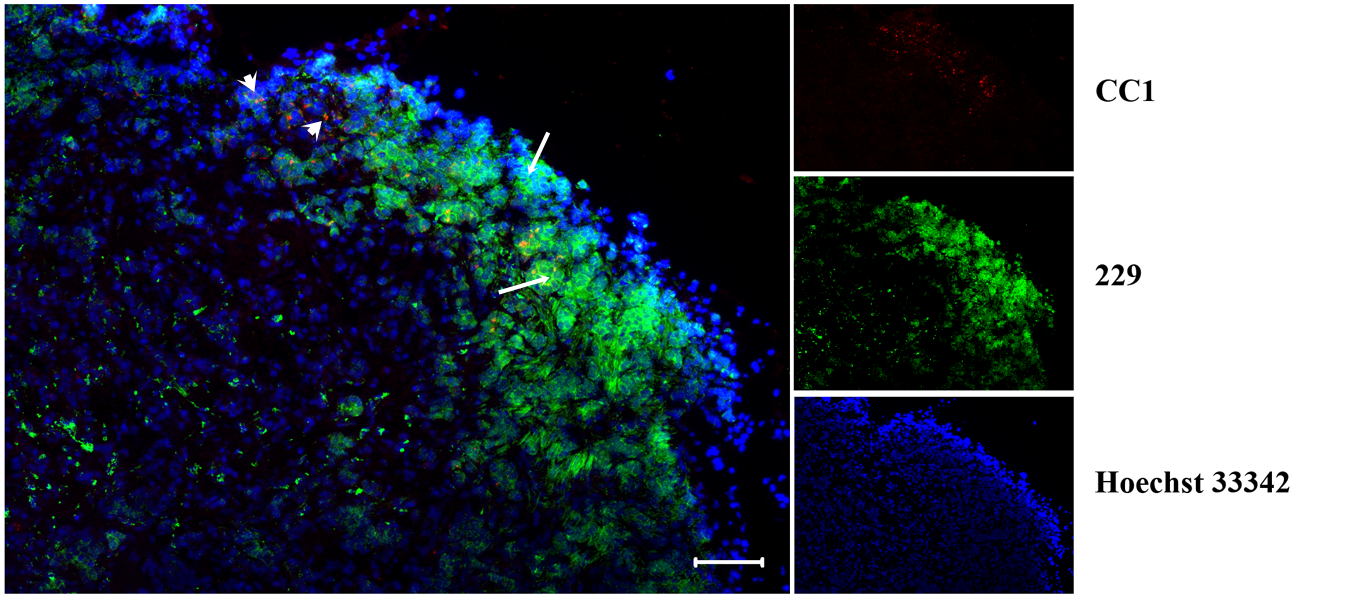

Supplement: Additional file 6: Figure S5 — Expression pattern of mouse CEACAM1 in vivo. Immunofluorescence staining of mouse CEACAM1 expressing cells from MDA-MB-468 tumor tissue. CEACAM1 expressing cells from MDA-MB-468 tumor tissue were treated with antibodies directed to the ectodomain shown in red (CC1, mouse antibody 1:200; shown with white arrowheads) or the -L cytoplasmic tail of human CEACAM1 by comparison and shown in green (229 rabbit antibody 1:200; shown with white arrows) followed by secondary antibodies (goat anti-rabbit, Alexa Fluor 488 labeled and goat anti-mouse, Alexa Fluor 555 labeled; 1:200). For nuclei staining, Hoechst 33342 (blue) was used at final concentration of 1 μg/ml. Scale bar, 100 μm. [file 1476-4598-13-64-S6.tiff]
